# Stem Cell Therapy Using Bone Marrow-Derived Muse Cells Repairs Radiation-Induced Intestinal Injury Through Their Intestine-Homing via Sphingosine Monophosphate-Sphingosine Monophosphate Receptor 2 Interaction

**DOI:** 10.1016/j.adro.2024.101565

**Published:** 2024-07-09

**Authors:** Taichi Miura, Junko Kado, Hirotoshi Takiyama, Mitsuko Kawano, Asako Yamagiri, Shoko Nishihara, Shigeru Yamada, Fumiaki Nakayama

**Affiliations:** a Regenerative Therapy Research Group, Department of Radiation Regulatory Science Research, National Institute of Radiological Sciences (NIRS), National Institutes for Quantum Science and Technology (QST), Chiba, Japan; b QST Hospital, National Institutes for QST, Chiba, Japan; c Glycan and Life Systems Integration Center (GaLSIC), Soka University, Hachioji, Tokyo, Japan; d Laboratory of Cell Biology, Department of Biosciences, Graduate School of Science and Engineering, Soka University, Hachioji, Tokyo, Japan; e Donation Course of Advanced Regenerative Plastic Surgery, Chiba University Graduate School of Medicine, Chiba, Japan

## Abstract

**Purpose:**

There is still no effective treatment for the gastrointestinal side effects of radiation therapy. Multilineage-differentiating stress-enduring (Muse) cells are tissue stem cells that have the ability to spontaneously home in on injured tissues and repair them. Several clinical trials have shown that stem cell therapy using human bone marrow-derived Muse (hBM-Muse) cells is effective in treating various diseases, but it is not known whether they are effective in treating radiation-induced intestinal injury. In this study, we investigated whether hBM-Muse cells are homing to the radiation-damaged intestine and promote its repair.

**Methods and Materials:**

hBM-Muse cells were injected into the tail vein of mice 2 hours after high-dose total body irradiation. Then, homing analysis, crypt assay, bromodeoxyuridine assay, Terminal deoxynucleotidyl transferase-mediated deoxyuridine triphosphate nick end labeling (TUNEL) assay, immunostaining, and survival time measurements were performed. In addition, we analyzed the expression of sphingosine monophosphate (S1P), a Muse cell-inducing factor, in the mouse small intestine after irradiation. Finally, we investigated whether the administration of JTE-013, an S1P receptor 2-specific antagonist, inhibits hBM-Muse cells homing to the injured intestine.

**Results:**

S1P expression increased in mouse intestine after irradiation, with hBM-Muse cells homing in on the injured intestine. Injection of hBM-Muse cells after radiation exposure significantly increased the number of crypts, proliferating cells in the crypts, and small intestinal component cells such as intestinal stem cells inhibited radiation-induced apoptosis and prolonged mouse survival. Treatment with JTE-013 significantly inhibited intestinal homing and therapeutic effects of hBM-Muse cells. These findings indicate that hBM-Muse cells homed in on the injured intestine through the S1P-S1P receptor 2 interaction to exert therapeutic effects on the radiation-induced intestinal injury.

**Conclusions:**

This study indicates that hBM-Muse cells are effective in treating radiation-induced intestinal injury, suggesting that hBM-Muse cell-based stem cell therapy has the potential to overcome gastrointestinal side effects that limit the indications for radiation therapy.

## Introduction

Within the gastrointestinal tract, the small intestine is particularly sensitive to radiation. When the small intestine is exposed to high-dose radiation, radiosensitive intestinal stem cells (ISCs) are lost, and the supply of functional cells to the villi is disrupted, resulting in a failure of epithelial structure.^1^ Additionally, symptoms such as diarrhea, hemorrhage, infection, and sepsis occur, leading to subacute death.^1^ These acute symptoms are termed gastrointestinal syndrome and represent a significant clinical problem that still lacks effective treatment. Thus, establishing a treatment for gastrointestinal syndrome is necessary to reduce the side effects of abdominal radiation therapy and to treat patients accidentally exposed to high doses of radiation.

Multilineage-differentiating stress-enduring (Muse) cells were first reported in 2010 as stage-specific embryonic antigen-3 (SSEA-3)-positive endogenous pluripotent-like stem cells and are found in bone marrow, peripheral blood, fat, umbilical cord, and the connective tissues of various organs.^2^^,^^3^ Additionally, Muse cells are present in and easily isolated from commercially available human bone marrow (hBM)-derived mesenchymal stem cells (hBM-MSCs), human adipose-derived MSCs, and human fibroblasts.^3^^,^^4^ Muse cells home in on injured tissues to exert a variety of therapeutic effects, including the promotion of cell growth, angiogenesis, and antiapoptosis.^3^^,^^5^ Previous studies have shown that sphingosine monophosphate (S1P), one of the universal injury signals secreted by injured tissues, interacts with S1P receptor 2 (S1PR2) on Muse cells, allowing Muse cells to home in on the injured tissue^5^^,^^6^ However, it is reported that non-MUSE cells (SSEA-3⁻ MSCs) lack the homing ability to target injured tissues through S1P-S1PR2 interactions.^5^^,^^6^ Furthermore, Muse cells are nontumorigenic and avoid host immune rejection, attracting attention as a new therapeutic tool in the field of regenerative therapy research.^3^^,^^5^ To date, most Muse cell therapy studies used hBM-derived Muse (hBM-Muse) cells, with clinical trials conducted for stroke, acute myocardial infarction, epidermolysis bullosa, spinal cord injury, neonatal hypoxic-ischemic encephalopathy, and amyotrophic lateral sclerosis.^3^^,^^5^ Recently, human Muse cells isolated from Wharton's jelly matrix of the umbilical cord were shown to have therapeutic effects against radiation-induced intestinal injury.^7^ However, the details of the hBM-Muse cell therapeutic effects on radiation-induced intestinal injury and the Muse cell homing mechanism to the injured intestine remain unclear.

In this study, we investigated the therapeutic effects of hBM-Muse cells on radiation-induced intestinal injury and the homing mechanism to the injured intestine using a mouse model.

## Methods and Materials

### Culture of hBM-MSCs

hBM-MSCs purchased from Lonza (catalog number: PT-2501) were cultured in low-glucose Dulbecco's modified Eagle's medium (Thermo Fisher Scientific) containing 10% fetal bovine serum (Serana Europe), 0.1 mg/mL kanamycin sulfate (Nacalai Tesque Inc), and 1 ng/mL human FGF-2 (ORF Genetics) at 37 °C in 95% air and 5% CO_2_. hBM-Muse cells and SSEA-3⁻ cells (non-Muse cells) were isolated from 5 passages of hBM-MSCs.

### Isolation of hBM-Muse cells

hBM-Muse cells and SSEA-3⁻ cells were isolated by fluorescence-activated cell sorting (FACS), as described previously.^4^ Briefly, hBM-MSCs were incubated with 5 μg/mL rat anti–SSEA-3 antibody (BioLegend) or 5 μg/mL Rat IgM Isotype Control (BioLegend) for 1 hour on ice and then incubated with 10 μg/mL FITC-conjugated anti-rat IgM (Jackson ImmunoResearch) for 1 hour on ice. hBM-Muse cells (high SSEA-3 expressing cells) and SSEA-3⁻ cells were isolated using a FACSAria II SORP Cell Sorter (Becton Dickinson). Harvested hBM-Muse cells or SSEA-3⁻ cells were suspended in CELLBANKER 1 plus (ZENOGEN PHARMA) and stored frozen at −80 °C until use. When frozen cells were used for experiments such as injections, the cells were thawed in a 37 °C water bath, and prewarmed low-glucose Dulbecco's modified Eagle's medium (Thermo Fisher Scientific) containing 10% fetal bovine serum (Serana Europe) and 0.1 mg/mL kanamycin sulfate (Nacalai Tesque Inc) was added.

### Mice

All animal experiments were performed at National Institute of Radiological Sciences (NIRS). All animal experiments were carried out with the permission of and under the regulation of the NIRS Institutional Animal Care and Use Committee (permit number 18-1020-2), and all experiments were performed in accordance with relevant guidelines and regulations. Male BALB/c mice (7 weeks of age, 23-27 g) were purchased from Clea Japan. All mice were acclimated for 7 days. They were housed under specific pathogen-free conditions. The animal rooms were maintained at 22 to 24 °C with a 12:12-hour light-dark schedule. Three to 5 mice were housed in a cage with a pellet diet and water available ad libitum. Eight-week-old mice were given single total body irradiation (TBI) of 10 or 12 Gy of γ-rays at a dose rate of approximately 0.40 Gy/min using a ^137^C source (Gammacell 40; Atomic Energy of Canada). Mice were irradiated bilaterally in groups of up to 12 mice without anesthesia in an appropriate position using a cylindrical Plexiglas container. When JTE-013, an S1PR2-specific antagonist, was administered, 4 mg/kg of JTE-013 (Cayman Chemical) was injected intraperitoneally in mice immediately after irradiation (IR).

Two hours after IR, hBM-Muse cells (or fluorescently labeled hBM-Muse cells) or SSEA-3⁻ cells (or fluorescently labeled SSEA-3⁻ cells) were injected into the tail vein of BALB/c mice at a concentration of 5 × 10^5^ cells in 200 μL of sterile saline. Controls received a tail vein injection of the same volume of sterile saline. Tail vein injections were performed using a 27-gauge needle, and no immunosuppressive drugs were administered to the mice. As previously reported,^8^ bromodeoxyuridine (BrdU) labeling reagent (Sigma-Aldrich) was injected intraperitoneally at 10 mL/kg 2 hours before sampling.

### Histology and immunostaining

As previously reported,^9^ mice were euthanized by cervical dislocation at 24 hours, 48 hours, and 3.5 days after IR, and approximately 11.2 to 14.0 cm of jejunum was removed from each mouse, which was cut into 10 portions, fixed in 10% buffered formalin, embedded in paraffin, and paraffin sections were prepared at 3 μm thickness. The crypt assay using jejunum at 3.5 days post-IR was performed in the same manner as previously reported.^9^ In 10 portions of hematoxylin-eosin–stained cross-sections of the jejunum per mouse, the number of crypts composed of more than 10 cells was counted under a microscope to assess the number of crypts per circumference. The average number of crypts was divided by the average number in non-IR saline-treated control mice for normalization.

Immunohistochemical staining was performed using the ImmPRESS HRP Goat Anti-Rabbit IgG Polymer Detection Kit (Vector Laboratories) and the Mouse on Mouse ImmPRESS HRP Polymer Kit (Vector Laboratories) according to the manufacturer's protocol. Sections were incubated overnight at 4 °C with the primary antibodies listed in Table E1. The DAB Substrate Kit (Vector Laboratories) was used for color development, and the number of stained cells was counted under a microscope.

### Statistical analysis

Unpaired 2-tailed Student's *t* test and one-way analysis of variance followed by Tukey's test were performed to compare 2 and 3 or more groups, respectively. Asterisks denote statistical significance (*P < .05; **P < .01). Statistical significance for Kaplan-Meier survival analysis was calculated using the log-rank test, with *P < .05 considered significant. Error bars represent SD.

Other experimental procedures are detailed in Appendix E1.

## Results

### Isolation of hBM-Muse cells from hBM-MSCs

**Figure 1:**
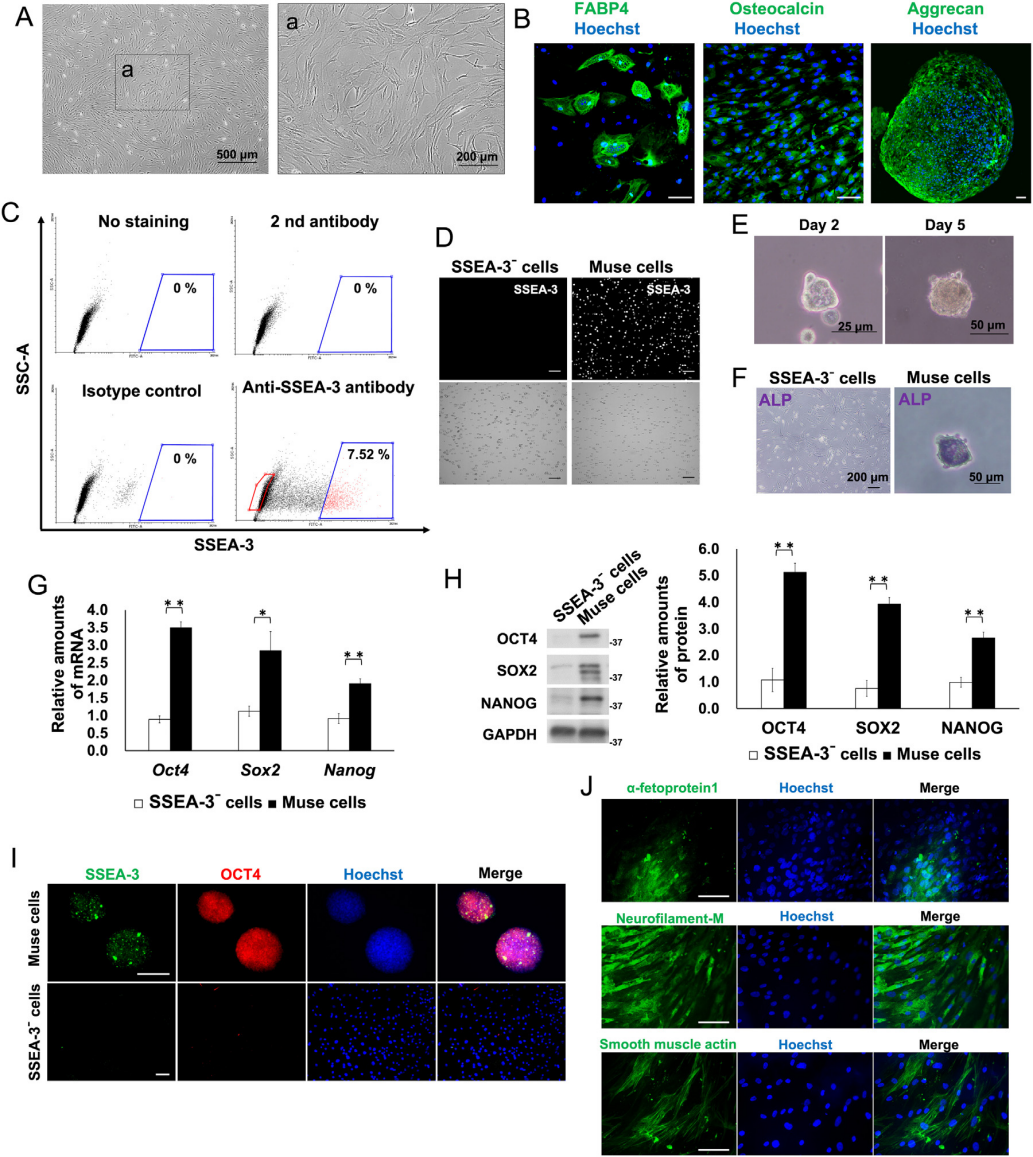
Isolation of human bone marrow (hBM)-derived multilineage-differentiating stress-enduring (hBM-Muse) cells from hBM-derived mesenchymal stem cells (hBM-MSCs). (A) Morphology of hBM-MSCs at 5 passages. (B) Immunostaining using antibodies against FABP4 (adipocyte marker), osteocalcin (osteocyte marker), and aggrecan (chondrocyte marker). These cells were differentiated from 5 passages of hBM-MSCs. Nuclei were stained with Hoechst. Scale bars, 100 μm. (C) Representative flow cytometry plots showing the percentage of hBM-Muse cells (stage-specific embryonic antigen-3 [SSEA-3]^+^ cells) isolated from hBM-MSCs. Blue and red gates indicate hBM-Muse cells and SSEA-3⁻ cell populations, respectively. Blue positive gates were determined using each sample immunostained with FITC-conjugated anti-rat IgM alone or together with Rat IgM isotype control. (D) Immunostaining with an antibody against SSEA-3 in hBM-Muse cells and SSEA-3⁻ cells after isolation. Scale bars, 200 μm. (E) Morphology of hBM-Muse cell clusters at days 2 and 5 after single-cell suspension culture. (F) Alkaline phosphatase (ALP) staining of hBM-Muse cell clusters and SSEA-3⁻ cells. (G) Real-time polymerase chain reaction analysis of *Oct4, Sox2*, and *Nanog* messenger (m)RNA expression in hBM-Muse cells. The amounts of each mRNA were normalized to that of *Gapdh* mRNA and are shown relative to SSEA-3⁻ cells. (H) Western blot analysis of OCT4, SOX2, and NANOG expression in hBM-Muse cells. Histograms show the mean densitometric readings ± SD of each undifferentiated marker/GAPDH after normalization against the levels in SSEA-3⁻ cells. (I) Immunostaining using antibodies against SSEA-3 and OCT4 in hBM-Muse cells and SSEA-3⁻ cells. Scale bars, 100 μm. (J) Immunostaining using antibodies against α-fetoprotein 1 (endoderm marker), neurofilament-M (ectoderm marker), and smooth muscle actin (mesoderm marker) in cells differentiated from hBM-Muse cells. Scale bars, 100 μm. Representative images are shown, and values were obtained from 3 independent experiments. Abbreviations: SSC-A = side scatter area.

Previous reports have shown the isolation of hBM-Muse cells as high SSEA-3 expressing cells from commercially available hBM-MSCs.^4^ In this study, hBM-Muse cells were isolated using hBM-MSCs at the fifth passage (Fig. 1A), and the differentiation potential of hBM-MSCs into adipocytes, osteocytes, and chondrocytes was confirmed prior to isolation (Fig. 1B). hBM-Muse cells were isolated by FACS using anti–SSEA-3 antibody with cells not expressing SSEA-3 (SSEA-3⁻ cells) isolated as negative controls (Fig. 1C, D). The percentage of hBM-Muse cells (high SSEA-3 expressing cells) in hBM-MSCs was 7% to 8%, which is consistent with previous reports.^4^^,^^7^ Isolated hBM-Muse cells formed alkaline phosphatase-positive cell clusters by single-cell suspension culture, whereas SSEA-3⁻ cells did not exhibit these characteristics (Fig. 1E, F). The expression of undifferentiated markers such as OCT4, SOX2, and NANOG was significantly higher in hBM-Muse cells than in SSEA-3⁻ cells (Fig. 1G-I). Additionally, hBM-Muse cells differentiated into α-fetoprotein 1 (endoderm marker), neurofilament-M (ectoderm marker), and smooth muscle actin (mesoderm marker)-positive cells (Fig. 1J). These results are consistent with previously reported Muse cell characteristics.^4^

### hBM-Muse cells have high therapeutic efficacy against IR-induced intestinal injury

**Figure 2:**
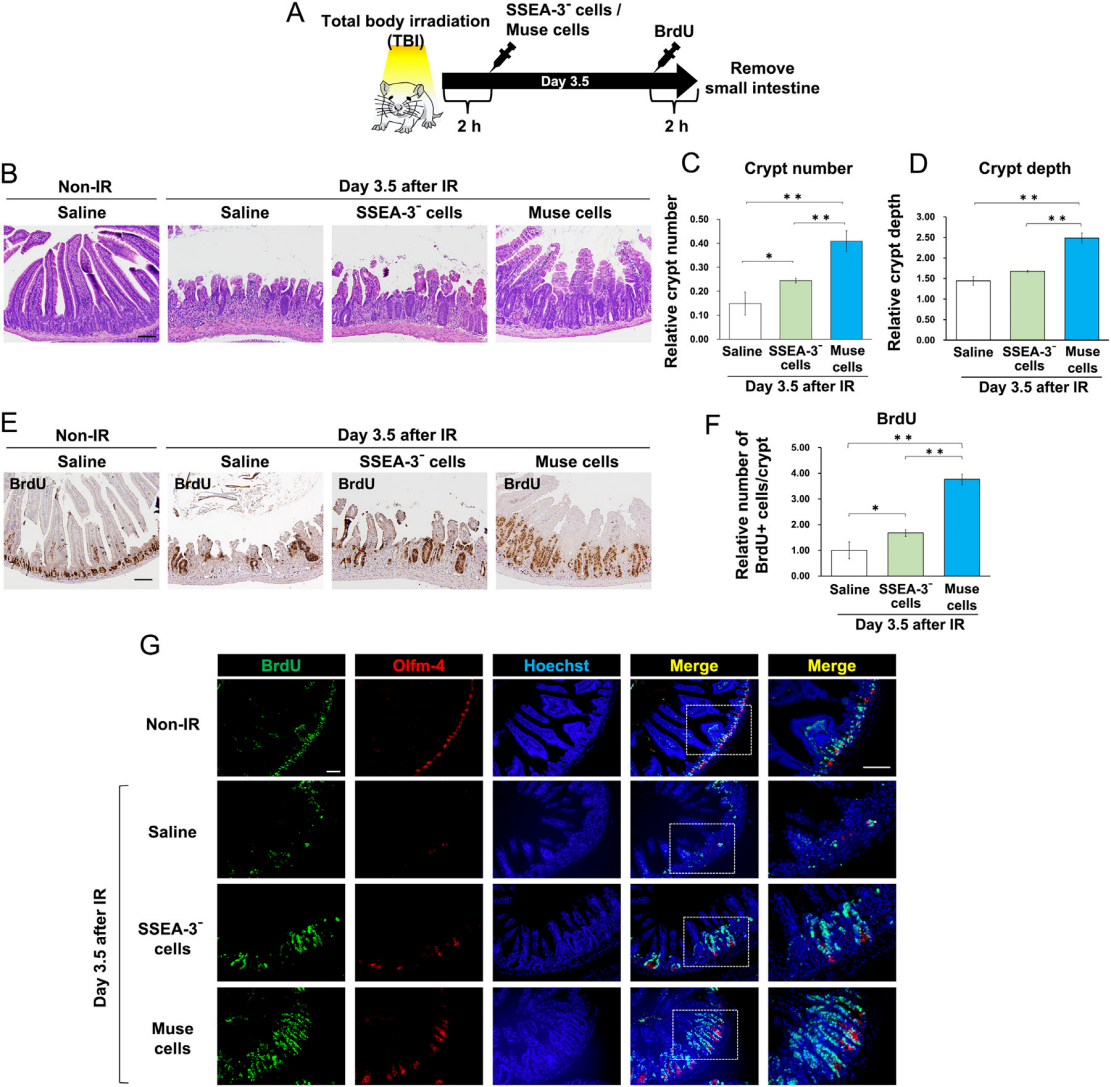
Human bone marrow-derived multilineage-differentiating stress-enduring (hBM-Muse) cells increase the number of crypts and proliferating cells in the crypts. (A) The scheme of the in vivo study. (B) Hematoxylin-eosin staining of the small intestine of hBM-Muse cell-, stage-specific embryonic antigen-3 (SSEA-3)⁻ cell-, and saline-treated mice at 3.5 days after 10 Gy irradiation (IR). (C, D) Histograms show the mean ± SD of the number of surviving crypts (C) and crypt depth (D) in mice at 3.5 days after 10 Gy IR after normalization against the values in non-IR mice. Values were obtained from 5 mice per group. (E) Immunostaining using an antibody against bromodeoxyuridine (BrdU) in the small intestine of mice at 3.5 days after 10 Gy IR. (F) Histograms show the mean ± SD of the number of BrdU+ cells/crypt in mice at 3.5 days after 10 Gy IR after normalization against the values of saline-treated mice. Values were obtained from 3 mice per group. (G) Immunostaining using antibodies against BrdU and Olfm-4 in the small intestine of mice at 3.5 days after 10 Gy IR. Nuclei were stained with Hoechst. Representative images of the immunostaining are shown. Scale bars, 100 μm. *Abbreviations:* TBI = total body irradiation.

To investigate the therapeutic effect of hBM-Muse cells on IR-induced intestinal injury, BALB/c mice were treated with high-dose (10 Gy) TBI with γ-rays, then hBM-Muse cells, SSEA-3⁻ cells, or saline were intravenously injected 2 hours after IR (Fig. 2A). The regenerative ability of the intestinal epithelium depends on the number of crypts and ISCs that are alive 3.5 days after IR.^10^ The number of crypts 3.5 days after IR was significantly increased in hBM-Muse cell-treated mice compared with saline- and SSEA-3⁻ cell-treated mice (Fig. 2B, C). Additionally, crypt depth was significantly increased in hBM-Muse cell-treated mice compared with saline- and SSEA-3⁻ cell-treated mice (Fig. 2D). BrdU analysis was performed to examine the number of proliferating cells (BrdU-positive cells) in the crypts. For hBM-Muse cell-treated mice, the number of BrdU-positive cells was significantly increased compared with those of saline- and SSEA-3⁻ cell-treated mice (Fig. 2E, F). Moreover, cells positive for the ISC marker Olfm-4 were localized in the crypt of hBM-Muse cell-treated mice (Fig. 2G).

**Figure 3:**
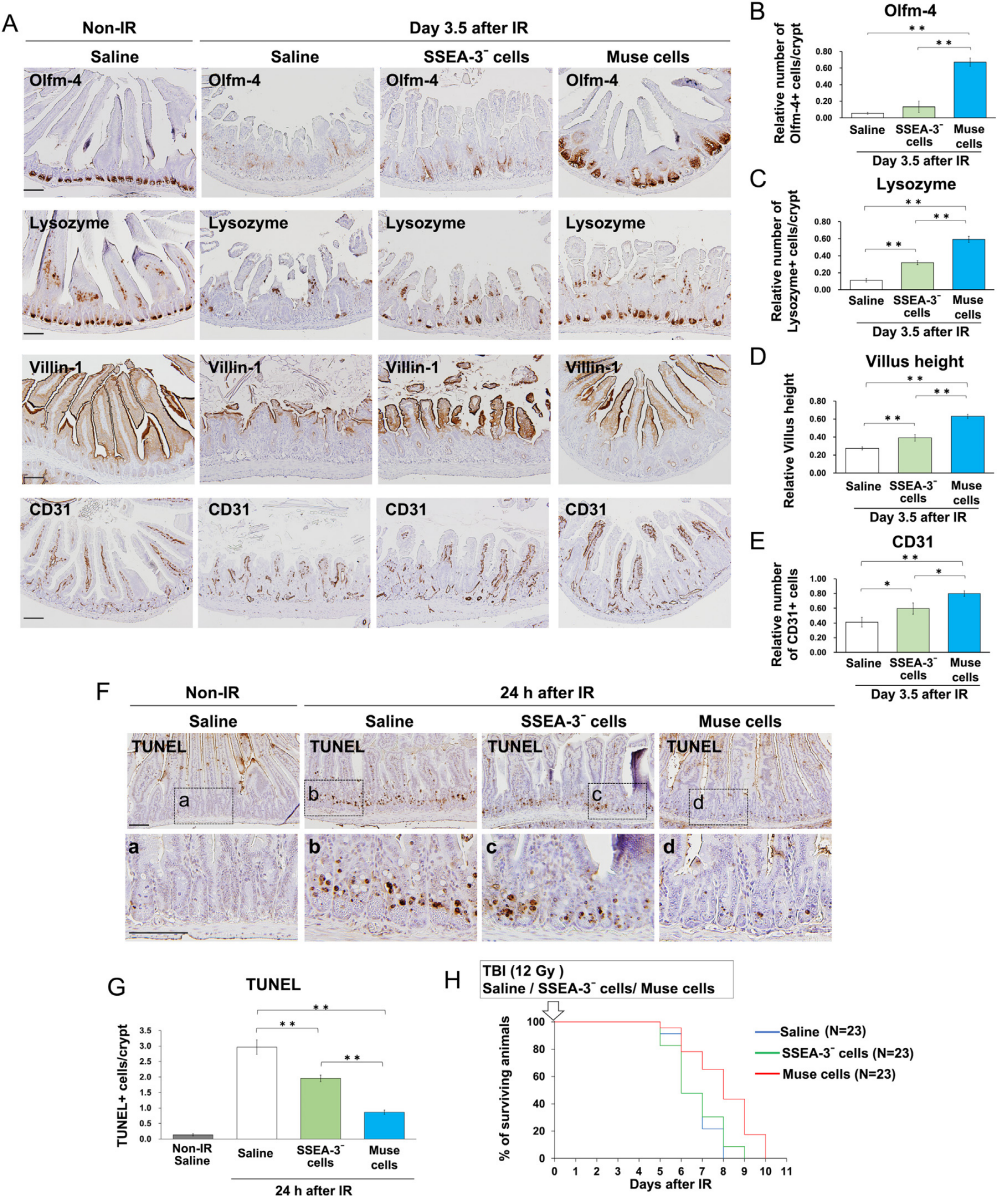
Human bone marrow-derived multilineage-differentiating stress-enduring (hBM-Muse) cells increased small intestinal constituent cells, inhibited apoptosis, and improved survival. (A) Immunostaining using antibodies against Olfm-4 (intestinal stem cell marker), lysozyme (paneth cell marker), Villin-1 (epithelial cell marker), and CD31 (endothelial cell marker) in the small intestine of hBM-Muse cell-, stage-specific embryonic antigen-3 (SSEA-3)⁻ cell-, and saline-treated mice at 3.5 days after 10 Gy irradiation (IR). (B-E) Histograms show the mean ± SD of Olfm-4+ cell number/crypt (B), lysozyme+ cell number/crypt (C), villus height (height of Villin-1–positive epithelium) (D), and CD31+ cell number (E) in the small intestine of mice at 3.5 days after 10 Gy IR after normalization against the values of non-IR mice. Values were obtained from 3 mice per group. (F) Terminal deoxynucleotidyl transferase-mediated deoxyuridine triphosphate nick end labeling (TUNEL) staining of the small intestine in hBM-Muse cell-, SSEA-3⁻ cell-, and saline-treated mice at 24 hours after 12 Gy IR. (G) Histograms show the mean ± SD of TUNEL+ cell number/crypt in the small intestine of mice at 24 hours after 12 Gy IR. Values were obtained from 4 mice per group. (H) Kaplan-Meier survival analysis of mice receiving hBM-Muse cells, SSEA-3⁻ cells, or saline 2 hours after 12 Gy total body irradiation (TBI). The significant difference between hBM-Muse cell-treated mice and saline- or SSEA-3⁻ cell-treated mice was *P* < .001. Representative images of the immunostaining and TUNEL staining are shown. Scale bars, 100 μm.

The cells constituting the small intestine, including ISCs, paneth cells, epithelial cells, and endothelial cells in the mouse small intestine 3.5 days after IR, were examined (Fig. 3A). The number of ISCs (Olfm-4–positive cells) and paneth cells (lysozyme-positive cells) in the crypt was significantly increased in hBM-Muse cell-treated mice compared with saline- and SSEA-3⁻ cell-treated mice (Fig. 3B, C). The height of villus positive for the epithelial cell marker Villin-1 was significantly greater in the small intestine of hBM-Muse cell-treated mice compared with saline- and SSEA-3⁻ cell-treated mice (Fig. 3D). These results indicate that intravenous injection of hBM-Muse cells increases the number of ISCs and crypts and promotes the repair of radiation-damaged small intestine.

The number of cells positive for the endothelial cell marker CD31 in the small intestine of mice began to decrease at 48 hours after 10 Gy IR (Fig. E1). In the small intestine of mice 3.5 days after IR, administration of hBM-Muse cells significantly increased the number of endothelial cells (CD31-positive cells) compared with administration of SSEA-3⁻ cells (Fig. 3E). These results suggest that injection of hBM-Muse cells promotes vascular regeneration in the small intestine of mice after IR.

To investigate IR-induced apoptosis in the small intestine, the Terminal deoxynucleotidyl transferase-mediated deoxyuridine triphosphate nick end labeling (TUNEL) assay was performed. The number of TUNEL-positive cells per crypt at 24 hours after IR was significantly reduced in SSEA-3⁻ cell-treated mice but even more significantly in hBM-Muse cell-treated mice (Fig. 3F, G). The lethal dose for 50% of the BALB/c mice 6 days after IR was 12 Gy TBI. hBM-Muse cells significantly prolonged survival after 12 Gy IR, whereas SSEA-3⁻ cells did not (Fig. 3H). Therefore, injection of hBM-Muse cells into mice after high-dose IR not only promotes intestinal regeneration but also inhibits radiation-induced apoptosis and prolongs survival.

These results demonstrate that hBM-Muse cells have a therapeutic effect on IR-induced intestinal injury and that their therapeutic effect is higher than that of SSEA-3⁻ cells.

### hBM-Muse cells highly express a variety of therapeutic factors that promote the regeneration of IR-induced intestinal injury

**Figure 4:**
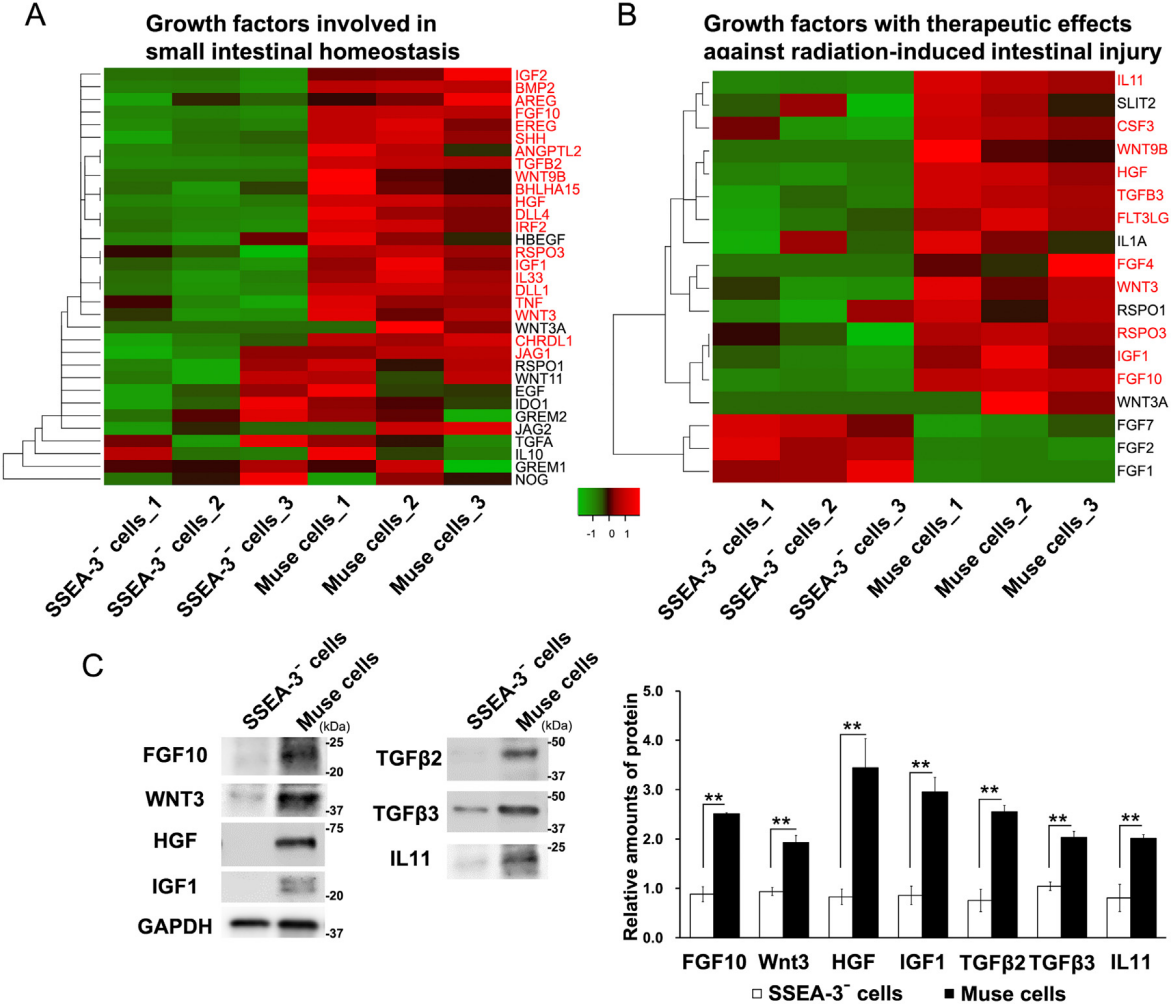
Human bone marrow-derived multilineage-differentiating stress-enduring (hBM-Muse) cells highly express a variety of therapeutic factors promoting regeneration of irradiation-induced intestinal injury. (A, B) The expression of various growth factors in hBM-Muse and stage-specific embryonic antigen-3 (SSEA-3)⁻ cells was examined by RNA sequencing (RNA-seq). Heatmap of the expression of growth factors involved in small intestinal homeostasis (A) and growth factors with therapeutic effects against radiation-induced intestinal injury (B). The expression values for each growth factor are based on Fragments Per Kilobase Million (FPKM) values. Significantly upregulated genes are identified in red. Data are shown as low-expression (green) and high-expression (red) genes. (C) Western blot analysis of fibroblast growth factor 10 (FGF10), wingless-type mouse mammary tumor virus integration site family member 3 (WNT3), hepatocyte growth factor (HGF), insulin-like growth factor 1 (IGF1), transforming growth factor beta2 (TGFβ2), TGFβ3, and interleukin-11 (IL-11) expression in hBM-Muse cells and SSEA-3⁻ cells. Histograms show the mean densitometric readings ± SD of each growth factor/GAPDH after normalization against the levels in SSEA-3⁻ cells. Representative images of Western blot analysis are shown, and values were obtained from 3 independent experiments.

We investigated the expression of growth factors involved in maintaining small intestinal homeostasis and promoting small intestinal regeneration in hBM-Muse cells by RNA sequencing (RNA-seq). Many of these factors, such as fibroblast growth factor 10 (FGF10), transforming growth factor beta2 (TGFβ2), hepatocyte growth factor (HGF), sonic hedgehog, and wingless-type mouse mammary tumor virus integration site family member 3 (WNT3) were found to be expressed more in hBM-Muse cells than in SSEA-3⁻ cells (Fig. 4A). Several growth factors are reported effective in treating IR-induced intestinal injury,^11^, ^12^, ^13^, ^14^ with many of these growth factors, such as FGF10, TGFβ3, HGF, WNT3, interleukin-11 (IL-11), and insulin-like growth factor 1 (IGF1), found to be expressed more in hBM-Muse cells than in SSEA-3⁻ cells (Fig. 4B). Among the genes significantly upregulated in hBM-Muse cells, 19 are reported to function in mice, including human FGF10, WNT3, HGF, IGF1, TGFβ2, TGFβ3, and IL-11.^11^, ^12^, ^13^, ^14^, ^15^, ^16^, ^17^, ^18^ Western blot analysis showed that the expression of human FGF10, WNT3, HGF, IGF1, TGFβ2, TGFβ3, and IL-11 proteins was significantly higher in hBM-Muse cells compared with SSEA-3⁻ cells (Fig. 4C). These results suggest that one reason for the greater efficacy of hBM-Muse cells than SSEA-3⁻ cells in treating radiation-IR intestinal injury is the higher expression of several growth factors involved in promoting small intestinal regeneration in hBM-Muse cells than in SSEA-3⁻ cells.

### hBM-Muse cells exert therapeutic effects by homing to sites of IR-induced intestinal injury through S1P-S1PR2 interaction

**Figure 5:**
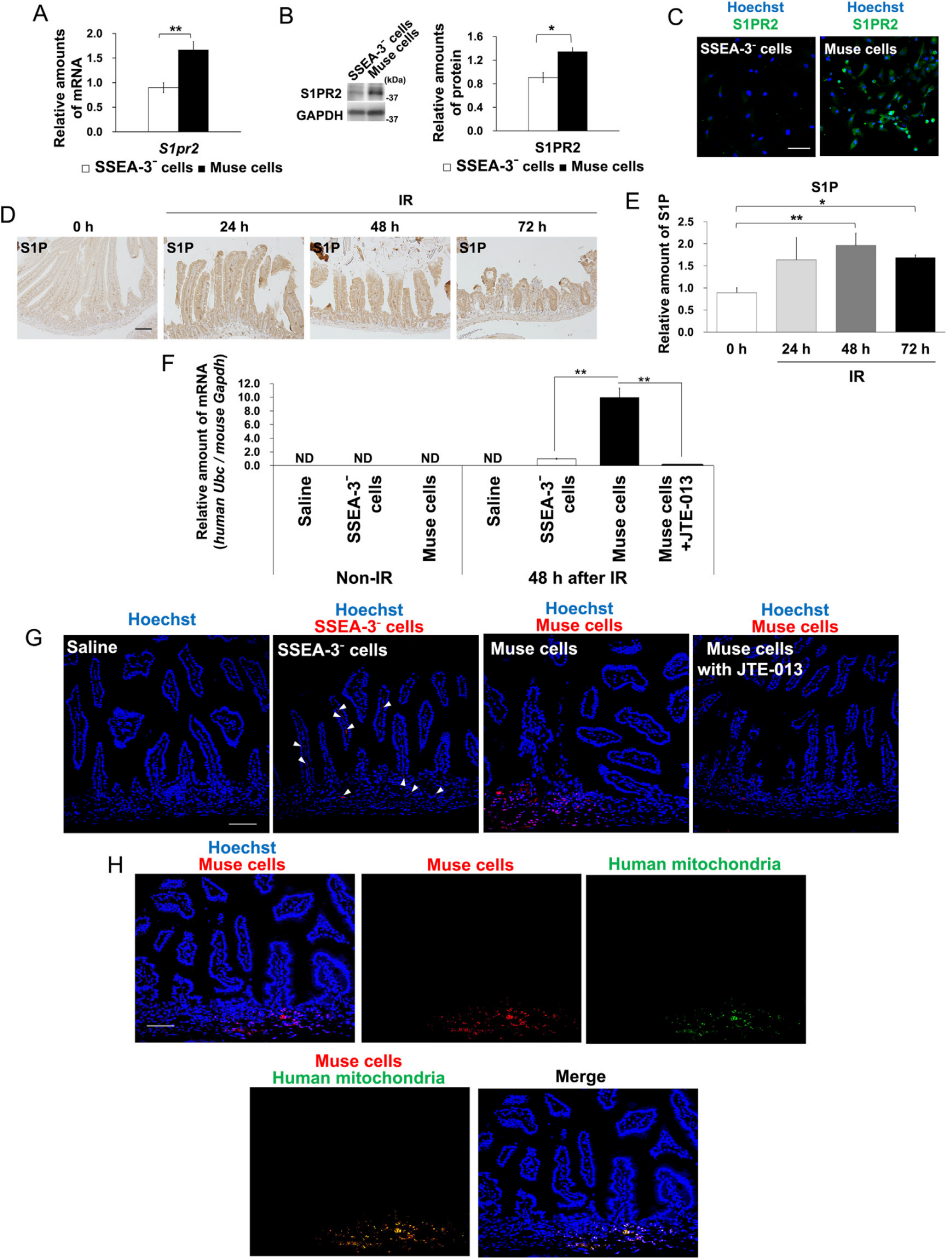
Human bone marrow-derived multilineage-differentiating stress-enduring (hBM-Muse) cells homing to sites of irradiation (IR)-induced intestinal injury through sphingosine monophosphate (S1P)-S1P receptor 2 (S1PR2) interaction. (A) Real-time polymerase chain reaction analysis of *S1pr2* messenger (m)RNA expression in hBM-Muse cells. The amounts of *S1pr2* mRNA were normalized to that of *Gapdh* mRNA and are shown relative to stage-specific embryonic antigen-3 (SSEA-3)⁻ cells. Values were obtained from 3 independent experiments. (B) Western blot analysis of S1PR2 expression in hBM-Muse cells and SSEA-3⁻ cells. Histograms show the mean densitometric readings ± SD of S1PR2/GAPDH after normalization against the levels in SSEA-3⁻ cells. Values were obtained from 3 independent experiments. (C) Immunostaining using an antibody against S1PR2 in hBM-Muse cells and SSEA-3⁻ cells. Scale bars, 100 μm. (D) Immunostaining using antibodies against S1P in the small intestine of mice at 24, 48, and 72 hours after 10 Gy IR. Scale bars, 100 μm. (E) Liquid chromatography-mass spectrometry/mass spectrometry (LC-MS/MS) analysis of S1P expression levels in the small intestine of mice at 24, 48, and 72 hours after 10 Gy IR. Histograms show the mean expression level of S1P ± SD after normalization against non-IR mice (0 hours). Values were obtained from 3 mice per group. (F) Real-time polymerase chain reaction analysis of human *Ubc* mRNA expression in the small intestine of hBM-Muse cells, SSEA-3⁻ cells, saline, and hBM-Muse cells + JTE-013 treated mice at 48 hours after 10 Gy IR. The amounts of human *Ubc* mRNA were normalized to that of mouse *Gapdh* mRNA and are shown relative to SSEA-3⁻ cell-treated mice at 48 hours after 10 Gy IR. Values were obtained from 3 mice per group. ND = not detected. (G) Immunostaining of fluorescently labeled hBM-Muse cells or fluorescently labeled SSEA-3⁻ cells in the small intestine of saline-, SSEA-3⁻ cells-, hBM-Muse cells-, and hBM-Muse cells + JTE-013-treated mice at 48 hours after 10 Gy IR. Arrowheads indicate fluorescently labeled SSEA-3⁻ cells. (H) Immunostaining to examine whether fluorescently labeled hBM-Muse cells in the small intestine of mice at 48 hours after 10 Gy IR are positive for human mitochondria. Nuclei were stained with Hoechst. Scale bars, 100 μm. Representative images of Western blot analysis and immunostaining are shown.

The homing ability of Muse cells to injured tissues involves the interaction between S1P, which is secreted by injured tissues, and S1PR2, which is highly expressed in Muse cells.^5^^,^^6^ In this study, S1PR2 expression was significantly higher in hBM-Muse cells than in SSEA-3⁻ cells (Fig. 5A-C). After 10 Gy IR, increased S1P expression was found in the small intestine of mice, with S1P expression reaching a maximum at 48 hours after IR (Fig. 5D, E).

Real-time polymerase chain reaction analysis using a human *Ubc*-specific TaqMan probe was performed to detect human-derived cells in the mouse small intestine after IR. In irradiated mice, hBM-Muse cells homed to the small intestine, but not in non-IR mice (Fig. 5F). SSEA-3⁻ cells underwent slight homing to the mouse small intestine after IR, but their levels were significantly lower than those of hBM-Muse cells. Furthermore, when hBM-Muse cells were injected into post-IR mice in combination with JTE-013, an S1PR2-specific antagonist, hBM-Muse cell homing to the small intestine was significantly inhibited. Next, we injected fluorescently labeled hBM-Muse cells into post-IR mice and found that fluorescently labeled hBM-Muse cells homed to the small intestine and that these cells were human mitochondria positive (Fig. 5G, H, and Fig. E2). The homing ability of fluorescently labeled SSEA-3⁻ cells to the small intestine was much lower than that of hBM-Muse cells to the small intestine. Additionally, treatment with JTE-013 strongly inhibited the homing of fluorescently labeled hBM-Muse cells into the small intestine after IR. These results indicated that hBM-Muse cells homing to the mouse small intestine after IR occurs through S1P-S1PR2 interaction. Human *Igf1, Hgf*, and *Wnt3* expression were highest in the small intestine of hBM-Muse cell-treated mice, and these expressions were suppressed by treatment with JTE-013 (Fig. E3). These results suggest that hBM-Muse cells that undergo homing to the mouse small intestine after IR express these growth factors in vivo. In this study, we did not obtain results indicating the differentiation of hBM-Muse cells or SSEA-3⁻ cells that underwent homing to the mouse small intestine after IR into small intestinal constituent cells such as epithelial or endothelial cells (data not shown).

**Figure 6:**
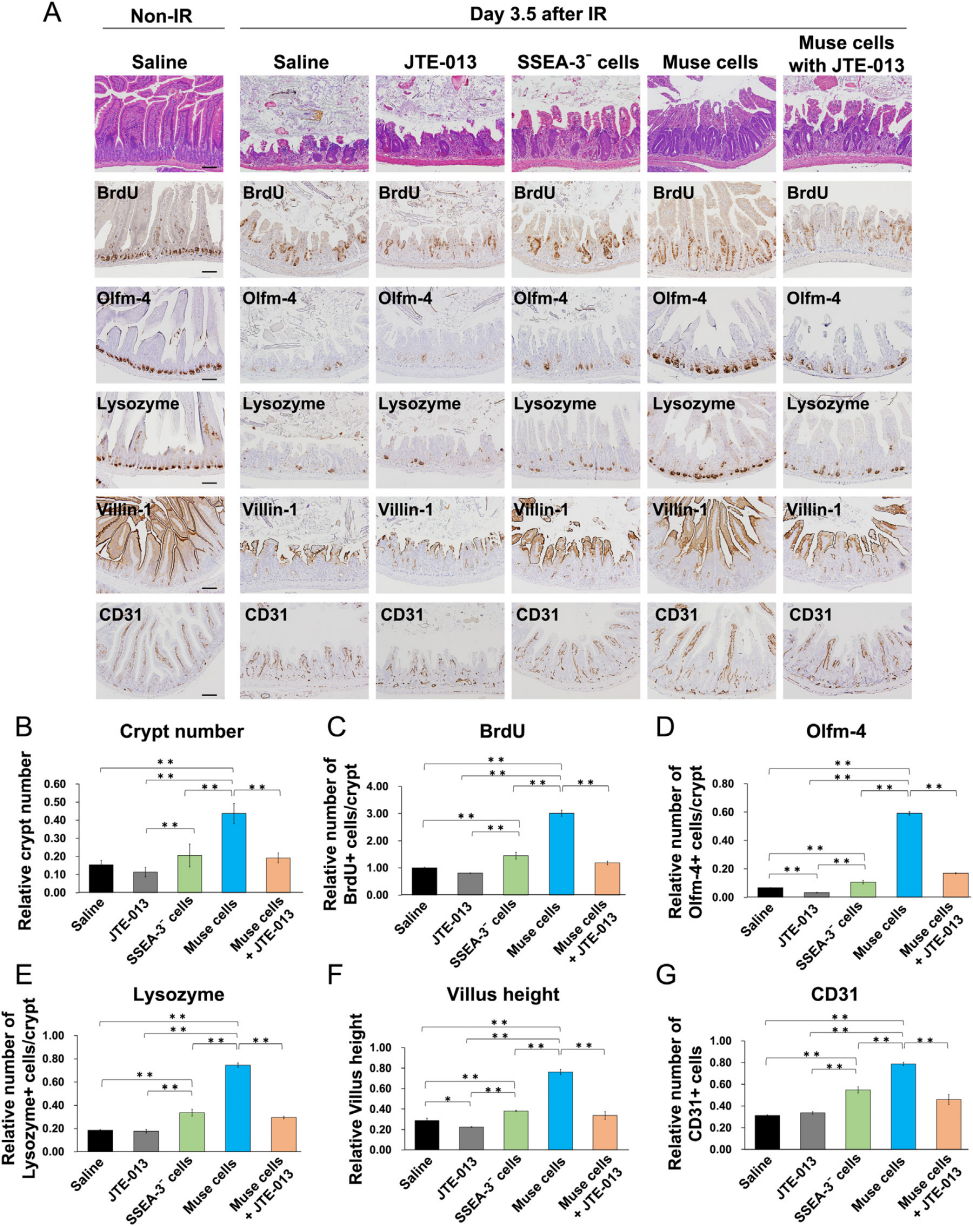
Human bone marrow-derived multilineage-differentiating stress-enduring (hBM-Muse) cells exert therapeutic effects by homing to sites of irradiation (IR)-induced intestinal injury. (A) Hematoxylin-eosin staining and immunostaining of the small intestine of saline-, JTE-013-, stage-specific embryonic antigen-3 (SSEA-3)⁻ cells-, hBM-Muse cells-, and hBM-Muse cells + JTE-013-treated mice at 3.5 days after 10 Gy IR. Scale bars, 100 μm. Representative immunostaining images are shown. (B) Histograms show the mean ± SD of the number of surviving crypts in mice at 3.5 days after 10 Gy IR after normalization against the values in non-IR mice. Values were obtained from 6 mice per group. (C) Histograms show the mean ± SD of the number of bromodeoxyuridine (BrdU)+ cells/crypt in the small intestine of mice at 3.5 days after 10 Gy IR after normalization against the values of saline-treated mice. Values were obtained from 3 mice per group. (D-G) Histograms show the mean ± SD of Olfm-4+ cell number/crypt (D), lysozyme+ cell number/crypt (E), villus height (F), and CD31+ cell number (G) in the small intestine of mice at 3.5 days after 10 Gy IR after normalization against the values of non-IR mice. Values were obtained from 3 mice per group.

Injection of hBM-Muse cells into mice after high-dose IR increased the number of crypts, proliferating cells in crypts, ISCs, paneth cells, villus height, and endothelial cells, all of which were significantly suppressed by the treatment with JTE-013 (Fig. 6A-G). These results indicated that injected hBM-Muse cells exert a therapeutic effect on IR-induced intestinal injury by homing to the small intestine after IR through S1P-S1PR2 interaction.

## Discussion

In this study, we demonstrated that administration of hBM-Muse cells significantly prolonged the survival of mice after high-dose TBI. However, as the survival analysis in this study was performed using an extreme condition of 12 Gy TBI without bone marrow transplantation, adverse events other than IR-induced intestinal injury may have affected the results of the survival analysis. For example, this study suggested that IR-induced bone marrow injury was not ameliorated by the administration of hBM-Muse cells (Fig. E4A). To clarify the relationship between hBM-Muse cells and intestinal death, survival analysis using an IR-induced intestinal injury model with partial abdominal IR is required.

Here, we demonstrated that injected hBM-Muse cells home to the small intestine after high-dose IR damage. However, hBM-Muse cells homing to mouse bone marrow after IR were not detected in this study (Fig. E4B). Injected Muse cells are reported to exert therapeutic effects by homing to various organs such as the skin, lungs, and brain.^3^^,^^5^ Despite the lack of reports detailing the homing of Muse cells to the large intestine, hBM-MSCs have been reported to home to the large intestine of mice.^19^ In addition to IR-induced intestinal injury, there is a need to develop treatments for IR-induced large intestine, skin, lung, and brain (nerve) injury, and human Muse cells may be effective in treating radiation injury to these tissues.

We showed that several growth factors, such as FGF10, WNT3, TGFβ3, IGF1, and IL-11, are highly expressed in hBM-Muse cells (Fig. 4B, C). However, this study did not clarify the mechanisms behind hBM-Muse cell-induced repair of IR-induced intestinal injury in mice in vivo. Previously, FGF10 was shown to promote recovery of the injured small intestine in a mouse model of IR-induced intestinal injury by stimulating the proliferation of small ISCs and epithelial cells.^11^ Additionally, TGFβ3, WNT3, and IGF1 have also been shown to have therapeutic effects against IR-induced intestinal injury in previous analyses using mouse or rat models.^12^, ^13^, ^14^ Our results suggest that hBM-Muse cells homing to the irradiated mouse small intestine secrete growth factors such as FGF10, WNT3, TGFβ3, and IGF1, which act in combination to promote small intestinal regeneration. Future studies are required to determine whether hBM-Muse cells homing to the site of IR-induced intestinal injury secrete these growth factors and whether these growth factors subsequently induce the repair of IR-induced intestinal injury. Additionally, this study showed that hBM-Muse cells inhibit radiation-induced apoptosis, but the mechanism behind the radiation-induced apoptosis inhibition remains unknown. Human IGF1 has previously been reported to inhibit radiation-induced apoptosis through activation of the Protein Kinase B (AKT) signaling pathway in the mouse small intestine.^14^ Human HGF is also reported to inhibit radiation-induced apoptosis.^20^ In this study, it is possible that these growth factors, which were highly expressed in hBM-Muse cells, inhibited radiation-induced apoptosis in the mouse small intestine. Additionally, human Muse cells are known to have potent antioxidant capacity,^21^ potentially scavenging IR-generated reactive oxygen species. These outcomes highlight the necessity of investigating the mechanisms controlling the administered hBM-Muse cells’ inhibition of radiation-induced apoptosis. Furthermore, Muse cells are reported to have anti-inflammatory and immunomodulatory effects in addition to the ability to secrete various growth factors.^3^^,^^5^ Therefore, an enormous amount of effort will be required to elucidate the therapeutic mechanism of Muse cells for IR-induced intestinal injury, and clarification of this research question is required to establish Muse cell therapies for IR-induced intestinal injury.

This study demonstrated that hBM-Muse cells are more effective than SSEA-3⁻ cells in the treatment of IR-induced intestinal injury. Additionally, SSEA-3⁻ cells were found to also have a therapeutic effect on IR-induced intestinal injury, albeit less than hBM-Muse cells. For example, when SSEA-3⁻ cells were administered to mice after IR, SSEA-3⁻ cells homed slightly into the small intestine, resulting in a significant increase in the number of crypts, paneth cells, and endothelial cells compared with controls (Figs. 2B and 3A). Moreover, SSEA-3⁻ cells significantly inhibited radiation-induced apoptosis (Fig. 3G). However, unlike hBM-Muse cells, administration of SSEA-3⁻ cells did not increase ISCs or prolong survival after IR (Fig. 3A, H). In this study, the therapeutic mechanism of SSEA-3⁻ cells in vivo was not clarified, and elucidating this for SSEA-3⁻ cells as well as hBM-Muse cells remains an important goal. Additionally, as SSEA-3 expression levels range widely in hBM-MSCs (Fig. 1C), it is also important to determine whether these correlate with the therapeutic effect on IR-induced intestinal injury.

In this study, we investigated the therapeutic effects of hBM-Muse cells using a mouse model of high-dose radiation injury. Therefore, for practical application of Muse cells, further analysis using protocols similar to those used in human treatment, such as multiple low-dose IR and partial abdominal IR, is needed. It is also necessary to determine whether administering Muse cells to mice long after IR (eg, 24 hours) has a therapeutic effect. In addition, it is important to examine potential changes in S1P expression in the human intestine after IR.

## Conclusions

This study demonstrated that intravenous injection of hBM-Muse cells promotes intestinal regeneration by homing to the site of IR-induced intestinal injury via S1P-S1PR2 interaction. Clinical trials of allogeneic hBM-Muse cell therapies for various diseases such as stroke and spinal cord injury have shown safety and high therapeutic efficacy. Therefore, hBM-Muse cells have high potential for clinical application in the treatment of radiation-induced intestinal injury, and further research on hBM-Muse cell function and therapeutic effects is necessary.

## Disclosures

None.

## Appendix Supplementary materials

Appendix E1_R_2 Image, application 1

## Footnotes

Supplementary material associated with this article can be found in the online version at doi:10.1016/j.adro.2024.101565.
