# Supplementary material for: Stem Cell Therapy Using Bone Marrow-Derived Muse Cells Repairs Radiation-Induced Intestinal Injury Through Their Intestine-Homing via Sphingosine Monophosphate-Sphingosine Monophosphate Receptor 2 Interaction
Source: Adv Radiat Oncol. 2024 Jul 9;9(9):101565. doi: 10.1016/j.adro.2024.101565 (PMC11345296; doi:10.1016/j.adro.2024.101565)
Supplement: Appendix E1_R_2 [file mmc1.pdf]

Supplementary Material for:

Stem cell therapy using bone marrow-derived Muse cells repairs radiation-induced intestinal injury through their intestine-homing via S1P-S1PR2 interaction

Supplementary Materials and Methods

Supplemental Table E1. Antibodies used for immunohistochemical staining

Supplemental Table E2. Antibodies used for immunofluorescent staining

Supplemental Table E3. Antibodies used for western blotting

Supplemental Table E4. TaqMan probes used for real-time PCR

Supplemental Table E5. Other materials used in this study

Supplementary Fig. E1. After high-dose IR, CD31-positive cells decreased in the small intestine of mice.

Supplementary Fig. E2. Fluorescently labelled hBM-Muse cells homing to the mouse small intestine after IR.

Supplementary Fig. E3. Human *Igf1*, *Hgf*, and *Wnt3* were highly expressed in the small intestine of hBM-Muse cell-treated mice after IR.

Supplementary Fig. E4. hBM-Muse cells did not home to the bone marrow of the mice after IR.

Supplementary Materials and Methods

Induction of differentiation of hBM-MSCs into adipocytes, osteocytes, and chondrocytes

Induction of hBM-MSC differentiation into adipocytes, osteocytes, and chondrocytes and detection of their respective differentiation markers was performed using the Human Mesenchymal Stem Cell Functional Identification Kit (R&D Systems, Minneapolis, MN) according to the manufacturer's protocol. Briefly, the cells were cultured for 2 to 3 weeks using the respective differentiation induction media included in the kit, and then FABP4 (adipocyte marker), Osteocalcin (osteocyte marker), and Aggrecan (chondrocyte marker) were detected by immunofluorescent staining.

Single-cell suspension culture, alkaline phosphatase (ALP) staining, differentiation into three germ layers

Single-cell suspension culture and ALP staining using hBM-Muse cells was performed as previously reported [1]. Briefly, to avoid adhesion of hBM-Muse cells to the culture dishes, the dishes were coated with a 3% Poly (2-hydroxethyl methacrylate) (poly-HEMA; Sigma-Aldrich) solution in 95% ethanol.

Isolated hBM-Muse cells were suspended in low-glucose DMEM (Thermo Fisher Scientific) containing 10% FBS (Serana Europe), 0.1 mg /ml kanamycin sulfate (Nacalai tesque) and 0.9% Methylcellulose (MethoCult H4100; StemCell Technologies, Vancouver, Canada), seeded onto poly-HEMA-coated culture dishes, and incubated for 5 days at 37°C in 95% air and 5% CO_2. MethoCult H4100 was used to avoid cell-to-cell adhesion between the hBM-Muse cells. ALP staining of hBM-Muse cell clusters at 5 days after single-cell suspension culture was performed using the Leukocyte Alkaline Phosphatase Kit (Sigma-Aldrich) according to the manufacturer's protocol.

Muse cell clusters are reported to spontaneously differentiate into three germ layers (endoderm, mesoderm, and ectoderm) when seeded in gelatin-coated culture dishes and cultured in adherent culture [1, 2]. As previously reported [1, 2], hBM-Muse cell clusters at 5 days after single-cell suspension culture were seeded into culture dishes pre-coated with 0.1% gelatin solution (Sigma-Aldrich) and cultured using low-glucose DMEM (Thermo Fisher Scientific) containing 10% FBS (Serana Europe) and 0.1 mg /ml kanamycin sulfate (Nacalai tesque) for 10 days. The expression of each differentiation marker, such as α-fetoprotein 1 (endoderm marker), Neurofilament-M (ectoderm marker), and Smooth muscle actin (mesoderm marker), was examined by immunofluorescence staining.

Immunofluorescent staining

For immunofluorescent staining of tissues, tissue sections were deparaffinized, autoclaved in 10 mM citrate buffer at 120°C for 15 min, and treated with 0.3% H_2O_2 to quench endogenous peroxidase activity. For immunofluorescent staining of cells, cells were fixed in 4% paraformaldehyde. Blocking and antibody dilution were performed using Blocking One Histo (Nacalai tesque) according to the manufacturer's protocol. Sections were incubated overnight at 4°C with the primary antibodies listed in Table E2, and after washing, sections were stained with the appropriate fluorescence-conjugated secondary antibody for 1 hour at room temperature. Nuclei were stained using Hoechst dye (Thermo Fisher Scientific). Sections were visualized using an FV3000 (Olympus, Tokyo, Japan).

Terminal deoxynucleotidyl transferase-mediated deoxyuridine triphosphate nick end labeling (TUNEL) assay

Apoptosis was evaluated with paraffin-embedded sections of the jejunum by TUNEL assay using an ApopTag Plus Peroxidase In Situ Apoptosis Detection Kit (Chemicon, Temecula, CA) according to the manufacturer's protocol. Briefly, tissue sections were deparaffinized and treated with 20 μg/ml Proteinase K and 3% H_2O_2 in PBS. After equilibration with buffer, tissue sections were treated with a

terminal deoxynucleotidyl transferase (TdT) enzyme to label the 3'-OH ends of DNA fragments with digoxigenin-nucleotides. Tissue sections were then incubated with a peroxidase-conjugated anti-digoxigenin antibody at room temperature for 30 min and color development was performed using DAB as a chromogen. After staining, at least 10 different portions of the jejunum of each mouse were photographed under a microscope and the number of TUNEL+ cells in each crypt was assessed. In this study, TUNEL+ cells in approximately 200-250 crypts per mouse were measured. The average number of TUNEL+ cells/crypt for each group was obtained from four mice in each group.

Fluorescent labeling of cells

Fluorescent labeling of hBM-Muse or SSEA-3^{-} cells was performed using the LuminiCell Tracker 670- Cell Labeling Kit (Sigma-Aldrich, St. Louis, MO) according to the manufacturer's protocol. Briefly, 400 μl 2 nM fluorescent labeling solution was added to 100 μl cell suspension (1×10^6 cells) and incubated at 37°C in 95% air and 5% CO_2, for 2 hours. The fluorescently labeled cells were then washed and used for injection into mice. In this Kit, the fluorescent dye enters the cell and the entire cell is labeled with intense fluorescence for a long term.

Measurement of S1P levels

After 10 Gy γ-ray TBI to BALB/c mice, the jejunum was removed at 24, 48, and 72 hours and stored frozen at -80°C. To examine the expression level of S1P in these tissues, LC-MS/MS analysis using TripleTOF 6600 (Sciex, Framingham, MA) and data analysis performed by Kazusa DNA Research Institute's Biomolecular Analysis Center (Chiba, Japan). The LC-MS/MS analysis in this study is a relative quantitative analysis. Three samples were analyzed from each group.

Western blotting

Cells were lysed with lysis buffer (50 mM Tris-HCl [pH 7.4], 150 mM NaCl, 1% Triton X-100, phosphatase inhibitor [PhosSTOP] [Roche Diagnostics, Mannheim, Germany], and protease inhibitors [cOmplete ULTRA] [Roche Diagnostics]). Ten micrograms of protein lysate from each sample were separated on a Mini-PROTEAN TGX gel (Bio-Rad Laboratories, Hercules, CA) and transferred to polyvinylidene fluoride membranes (Millipore, Burlington, MA). After blocking with bovine serum albumin (Thermo Fisher Scientific), membranes were incubated overnight at 4°C with the primary antibodies listed in Table E3. Then, membranes were washed and incubated with the appropriate HRP-conjugated secondary antibody, washed, and developed with ECL Select reagents (GE Healthcare,

Waukesha, WI).

Real-time PCR

Total RNA was isolated from cells and tissues using the RNeasy Mini Kit (QIAGEN, Venlo, Netherlands) and reverse transcribed using the SuperScript III First-Strand Synthesis System (Thermo Fisher Scientific) according to the manufacturer's protocol. Real-time PCR was performed on a LightCycler 480 (Roche Diagnostics) using the TaqMan probes (Thermo Fisher Scientific) listed in Table E4. The relative amount of each mRNA was normalized to the amount of *Gapdh* mRNA in the same sample. Real-time PCR analysis using the human *ubiquitin* (*Ubc*)-specific TaqMan probe (Hs00824723_m1) does not detect mouse *Ubc* (or other mouse genes). Therefore, to examine human-derived cells homing to the mouse small intestine, real-time PCR analysis using a human *Ubc*-specific TaqMan probe was performed.

RNA sequencing (RNA-seq)

RNA-seq library preparation, sequencing, mapping, and gene expression analysis were performed by DNAFORM (Kanagawa, Japan). Qualities of total RNA were assessed by Bioanalyzer (Agilent

Technologies, Santa Clara, CA) to ensure a RIN (RNA integrity number) over 7.0. After poly (A) + RNA enrichment by NEBNext Poly(A) mRNA Magnetic Isolation Module (New England BioLabs, Ipswich, MA), double-stranded cDNA libraries (RNA-seq libraries) were prepared using SMARTer Stranded Total RNA Sample Prep Kit HI Mammalian (Takara Bio, Shiga, Japan) according to the manufacturer's instruction. RNA-seq libraries were sequenced using paired end reads (50nt of read1 and 25nt of read2) on a NextSeq 500 instrument (Illumina, San Diego, CA). Obtained raw reads were trimmed and quality-filtered using the Trim Galore! (version 0.4.4), Trimmomatic (version 0.36), and cutadapt (version 1.16) software. Trimmed reads were then mapped to the human GRCh38.p13 genome using STAR (version 2.7.2b). Reads on annotated genes were counted using featureCounts (version 1.6.1). Fragments Per Kilobase Million (FPKM) values were calculated from mapped reads by normalizing to total counts and transcript.

Supplemental Table E1. Antibodies used for immunohistochemical staining

Antibody	Vendor	Product Code	Dilution	Incubation
Rabbit anti-Olfm-4 mAb	Cell Signaling Technology	39141S	1/1000	4℃, O/N
Rabbit anti-Lysozyme mAb	Abcam	ab108508	1/4000	4℃, O/N
Rabbit anti-Villin mAb	Abcam	ab130751	1/1000	4℃, O/N
Rabbit anti-CD31 mAb	Cell Signaling Technology	#77699S	1/100	4℃, O/N
Mouse anti-BrdU mAb	Cell Signaling Technology	#5292S	1/400	4℃, O/N
Mouse anti-S1P mAb	Echelon Biosciences	Z-P300	1/2000	4℃, O/N

Supplemental Table E2. Antibodies used for immunofluorescent staining

Antibody	Vendor	Product Code	Dilution	Incubation
Rabbit anti-Olfm-4 mAb	Cell Signaling Technology	39141S	1/500	4℃, O/N
Mouse anti-BrdU mAb	Cell Signaling Technology	#5292S	1/200	4℃, O/N
Rabbit anti-Oct4 mAb	Cell Signaling Technology	#2840	1/100	4℃, O/N
Rabbit anti-α-fetoprotein mAb	Cell Signaling Technology	#4448	1/100	4℃, O/N
Mouse anti-Smooth muscle actin mAb	BioLegend	904601	1/100	4℃, O/N
Mouse anti-Neurofilament-M mAb	Cell Signaling Technology	#2838	1/100	4℃, O/N
Rat anti-SSEA-3 mAb	Biolegend	330302	1/100	4℃, O/N
Rabbit anti-S1PR2 Ab	Proteintech	21180-1-AP	1/400	4℃, O/N
Anti-rabbit IgG, Alexa Fluor 488-linked Ab	Abcam	ab150077	1/200	r.t., 1 h
Anti-rabbit IgG, Alexa Fluor 568-linked Ab	Abcam	ab175471	1/200	r.t., 1 h
Anti-mouse IgG, Alexa Fluor 488-linked Ab	Abcam	ab150113	1/200	r.t., 1 h
Anti-rat IgM, Alexa Fluor 488-linked Ab	Jackson Immuno Research	112-545-075	1/200	r.t., 1 h
Mouse anti-human mitochondria mAb (Alexa Fluor 488)	Sigma-Aldrich	MAB1273A4	1/250	r.t., 1 h

Supplemental Table E3. Antibodies used for western blotting

Antibody	Vendor	Product Code	Dilution	Incubation
Rabbit anti-OCT4 mAb	Cell Signaling Technology	#2840	1/1000	4°C, O/N
Rabbit anti-SOX2 mAb	Cell Signaling Technology	#23064	1/1000	4°C, O/N
Mouse anti-NANOG mAb	Santa cruz	sc-293121	1/1000	4°C, O/N
Rabbit anti-S1PR2 Ab	Proteintech	21180-1-AP	1/1000	4°C, O/N
Mouse anti-FGF10 mAb	Santa cruz	sc-293208	1/1000	4°C, O/N
Mouse anti-WNT3 mAb	Santa cruz	sc-74537	1/1000	4°C, O/N
Mouse anti-HGFα mAb	Santa cruz	sc-374422	1/1000	4°C, O/N
Mouse anti-IGF1 mAb	Santa cruz	sc-74116	1/1000	4°C, O/N
Mouse anti-TGFβ2 mAb	Santa cruz	sc-374659	1/1000	4°C, O/N
Mouse anti-TGFβ3 mAb	Santa cruz	sc-166833	1/1000	4°C, O/N
Mouse anti-IL11 mAb	Santa cruz	sc-133063	1/1000	4°C, O/N
Mouse anti-GAPDH mAb	Santa cruz	sc-32233	1/1000	4°C, O/N
Anti-mouse IgG, HRP-linked Ab	Cell Signaling Technology	#7076	1/3000	r.t., 1 h
Anti-rabbit IgG, HRP-linked Ab	Cell Signaling Technology	#7074	1/3000	r.t., 1 h

Supplemental Table E4. TaqMan probes used for real-time PCR

Gene	Species	Vendor	TaqMan probe
Gapdh	Mus musculus	Thermo Fisher Scientific	Mm99999915_g1
Gapdh	Homo sapiens	Thermo Fisher Scientific	Hs99999905_m1
Oct4	Homo sapiens	Thermo Fisher Scientific	Hs01895061_u1
Nanog	Homo sapiens	Thermo Fisher Scientific	Hs02387400_g1
Sox2	Homo sapiens	Thermo Fisher Scientific	Hs01053049_s1
Ubc	Homo sapiens	Thermo Fisher Scientific	Hs00824723_m1
Igf1	Homo sapiens	Thermo Fisher Scientific	Hs01547656_m1
Hgf	Homo sapiens	Thermo Fisher Scientific	Hs00300159_m1
Wnt3	Homo sapiens	Thermo Fisher Scientific	Hs00902258_m1
S1pr2	Homo sapiens	Thermo Fisher Scientific	Custom TaqMan Gene Expression Assays (Assay ID:ARNKVPM)

Supplemental Table E5. Other materials used in this study

**Materials**	**Application**	**Vendor**	**Product Code**
Human Bone Marrow-derived Mesenchymal Stem Cells	Cell culture	Lonza	PT-2501
Low-glucose Dulbecco's Modified Eagle's Medium	Cell culture	Thermo Fisher Scientific	10567022
Fetal Bovine Serum (FBS)	Cell culture	Serana Europe	S-FBS-NL-015
Kanamycin Sulfate Solution	Cell culture	Nacalai Tesque	11981-04
Human FGF basic	Cell culture	ORF Genetics	IK0200
100 mm x 20mm culture dish	Cell culture	Corning	353003
Human Mesenchymal Stem Cell Functional Identification Kit	Cell culture	R&D Systems	SC006
Direct Heat Type CO_2/Multi-gas Incubator SCA Series	Cell culture	Astec	N/A
Bio Clean Bench MCV-B131F	Cell culture	PHC	N/A
Trypsin-EDTA (0.25%), phenol red	Cell culture	Invitrogen	25200072
FluoroBrite DMEM	Isolation of hBM-Muse cells	Invitrogen	A1896701
0.5mol/l-EDTA Solution (pH 8.0)	Isolation of hBM-Muse cells	Nacalai Tesque	06894-85
Bovine Serum Albumin (BSA)	Isolation of hBM-Muse cells	Sigma-Aldrich	A7030
Rat IgM Isotype control	Isolation of hBM-Muse cells	Biolegend	400801
D-PBS(-) without Ca and Mg, liquid	Isolation of hBM-Muse cells	Nacalai Tesque	14249-24
FACS Aria II SORP cell sorter	Isolation of hBM-Muse cells	BD biosciences	N/A

Continued

Materials	Application	Vendor	Product Code
CELLBANKER 1 plus	Cryopreservation	ZENOGEN PHARMA	ZR638
LuminiCell Tracker 670- Cell Labeling Kit	Fluorescent labeling of cells	Sigma-Aldrich	SCT011
3% Poly (2-hydroxethyl methacrylate)	Single-cell suspension culture	Sigma-Aldrich	P3932
Methylcellulose (MethoCult H4100)	Single-cell suspension culture	StemCell Technologies	#04100
Leukocyte Alkaline Phosphatase Kit	ALP staining	Sigma-Aldrich	86R
Gelatin	Differentiation into three germ layers	Sigma-Aldrich	G1890
Male BALB/c mice (7 weeks of age, 23-27 g)	Mouse	Clea Japan	N/A
Gammacell 40	Irradiation	Atomic Energy of Canada	N/A
27-gauge needle	Injection of cells	Terumo	NN-2719S
JTE-013	S1PR2-specific antagonist	Cayman Chemical	10009458
Dimethyl sulfoxide	Dilution of JTE-013	Sigma-Aldrich	D2650-5X10ML
BrdU	BrdU solution	Sigma-Aldrich	B5002-1G
FrdU	BrdU solution	Sigma-Aldrich	F0503
Hematoxylin	HE staining	Sakura Finetek Japan	9131-4P
Eosin	HE staining	Sakura Finetek Japan	9135-4P
ApopTag Plus Peroxidase In Situ Apoptosis Kit	TUNEL staining	Chemicon	S7101

Continued

Materials	Application	Vendor	Product Code
ImmPRESS HRP Goat Anti-Rabbit IgG Polymer Detection Kit	Immunohistochemical staining	Vector Laboratories	MP-7451-50
Mouse on Mouse ImmPRESS Peroxidase Polymer Kit	Immunohistochemical staining	Vector Laboratories	MP-2400
Hydrogen Peroxide (H_2O_2)	Histology and immunostaining	FUJIFILM	081-04215
Clear Plus	Histology and immunostaining	Falma	306-300-2
Ethanol	Histology and immunostaining	Falma	43102
Mounting agent, PARA mount-D	Histology and immunostaining	Falma	308-500-1
DAB Substrate Kit	Histology and immunostaining	Vector laboratories	SK-4100
Paraffin Embedding Machine, Tissue-Tek TEC Plus Dispensing Console	Histology and immunostaining	Sakura Finetek Japan	N/A
Paraffin Infiltrator, Tissue-Tek VIP 6	Histology and immunostaining	Sakura Finetek Japan	N/A
10% Formalin Neutral Buffer Solution	Histology and immunostaining	Muto Pure Chemicals	20214
Blocking One Histo	Immunofluorescent staining	Nacalai Tesque	06349-64
TrueVIEW Autofluorescence Quenching Kit	Immunofluorescent staining	Vector Laboratories	SP-8400
4%-Paraformaldehyde Phosphate Buffer Solution	Immunofluorescent staining	Nacalai Tesque	09154-14
Hoechst	Immunofluorescent staining	Thermo Fisher Scientific	H3570
Mounting agent, VECTASHIELD Vibrance Antifade Mounting Medium	Immunofluorescent staining	Vector Laboratories	H-1700

Continued

**Materials**	**Application**	**Vendor**	**Product Code**
Confocal Laser Scanning Microscope, FV3000	Immunofluorescent staining	Olympus	N/A
RNeasy Mini Kit	Real-time PCR	QIAGEN	74106
SuperScript III First-Strand Synthesis System	Real-time PCR	Thermo Fisher Scientific	18080051
LightCycler 480 System	Real-time PCR	Roche Diagnostics	N/A
LightCycler480 Probe Master	Real-time PCR	Roche Diagnostics	4707494001
Phosphatase Inhibitor (PhosSTOP)	Western blotting	Roche Diagnostics	4906837001
Protease Inhibitors (cOmplete ULTRA)	Western blotting	Roche Diagnostics	4693116001
Mini-PROTEAN TGX gel	Western blotting	Bio-Rad Laboratories	#4569036
Polyvinylidene Fluoride Membrane	Western blotting	Millipore	IPVH00010
Bovine Serum Albumin (BSA)	Western blotting	Sigma-Aldrich	A7030
Western Blotting Detection Reagent, ECL Select Reagent	Western blotting	GE Healthcare	RPN2235
LAS-4000mini	Western blotting	GE Healthcare	N/A

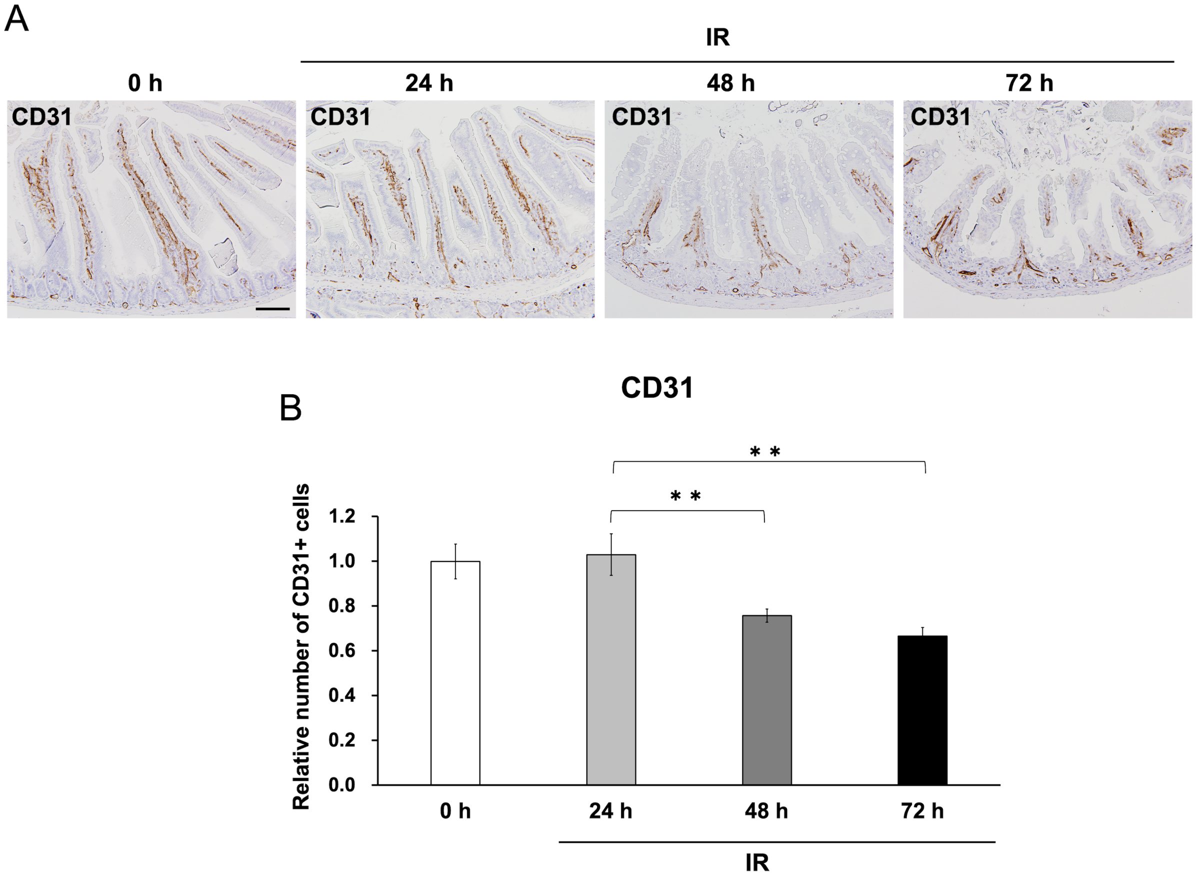

Supplementary Fig. E1. After high-dose IR, CD31-positive cell numbers decreased in the small intestine of mice.

(A) Immunostaining using antibodies against CD31 in the small intestine of mice at 24, 48, 72 h after 10 Gy IR. Scale bars, 100 μm. Representative images of immunostaining are shown. (B) Histograms show the mean ± SD of CD31+ cell number in the small intestine of mice at 24, 48, 72 h after 10 Gy IR, after normalization against the values of non-IR mice (0 h). Values were obtained from three mice per group.

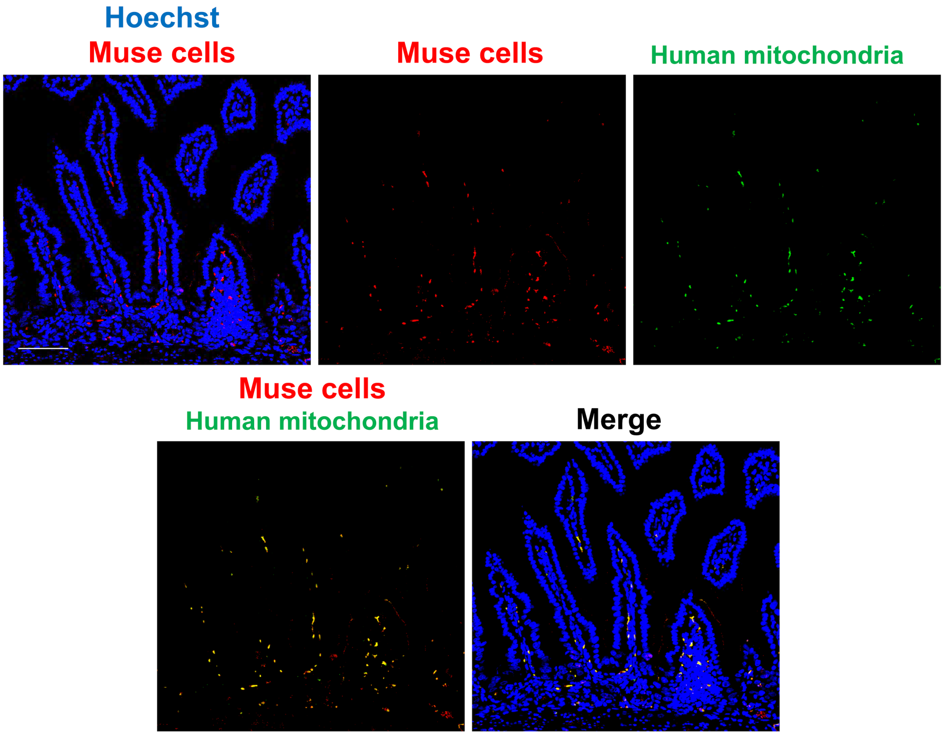

Supplementary Fig. E2. Fluorescently labeled hBM-Muse cells homing to the mouse small intestine after IR.

Immunostaining of fluorescently labeled hBM-Muse cells in the small intestine of hBM-Muse cells treated mice at 48 h after 10 Gy IR. Fluorescently labeled hBM-Muse cells in the small intestine of mice after IR are positive for human mitochondria. Nuclei were stained with Hoechst. Scale bars, 100 µm. Representative images of immunostaining are shown.

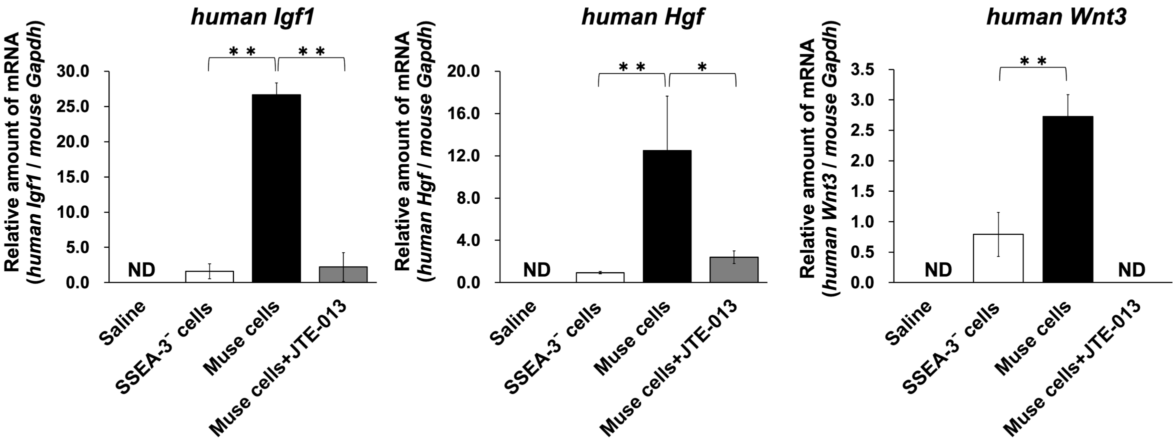

Supplementary Fig. E3. Human *Igf1*, *Hgf*, and *Wnt3* were highly expressed in the small intestine of hBM-Muse cell-treated mice after IR.

Real-time PCR analysis of human *Igf1*, *Hgf*, and *Wnt3* mRNA expression in the small intestine of hBM-Muse cells, SSEA-3$^-$ cells, saline, and hBM-Muse cells+JTE-013 treated mice at 48 h after 10 Gy IR. mRNA amounts were normalized to that of mouse *Gapdh* mRNA and are shown relative to SSEA-3$^-$ cell-treated mice at 48 h after 10 Gy IR. Values were obtained from three mice per group. Not detected: ND.

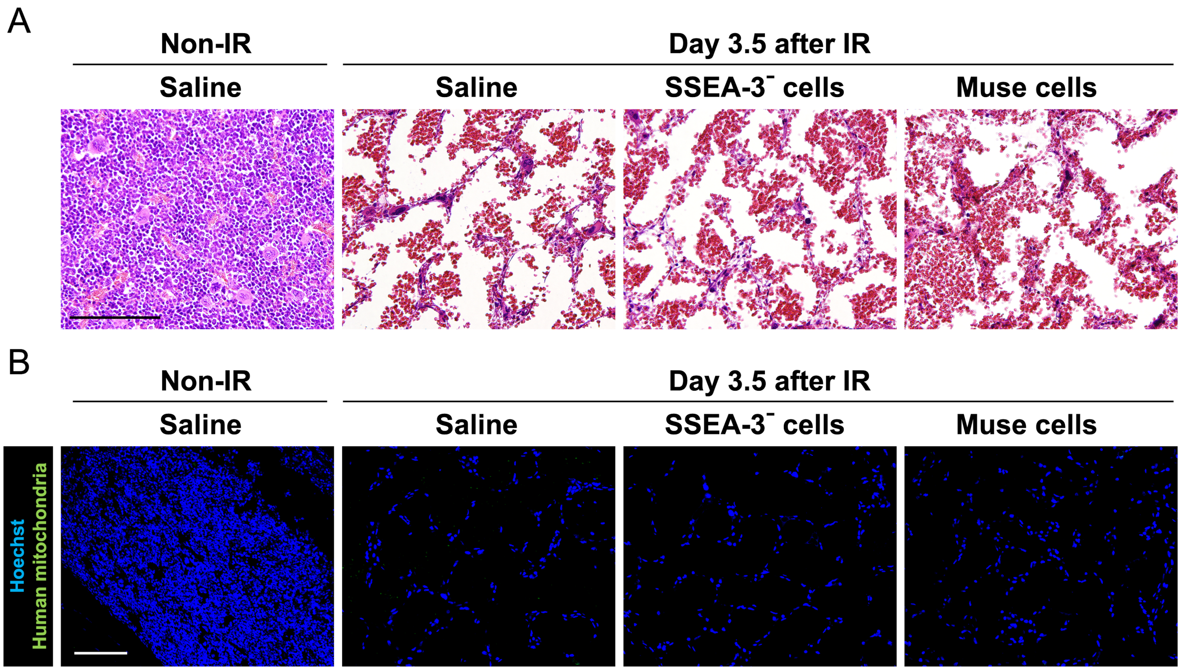

Supplementary Fig. E4. hBM-Muse cells did not home to the bone marrow of mice after IR.

(A) HE staining of the bone marrow of hBM-Muse cell-, SSEA-3⁻ cell-, and saline-treated mice at 3.5 days after 10 Gy IR. (B) Immunostaining using antibodies against human mitochondria in the bone marrow of saline, SSEA-3⁻ cells, and hBM-Muse cells treated mice at 3.5 days after 10 Gy IR. Nuclei were stained with Hoechst. Scale bars, 100 μm. Representative immunostaining images are shown.

References

1. Kuroda Y, Wakao S, Kitada M, et al. Isolation, culture and evaluation of multilineage-differentiating stress-enduring (Muse) cells. *Nat Protoc*. 2013;8:1391-1415. doi:10.1038/nprot.2013.076

2. Kuroda Y, Kitada M, Wakao S, et al. Unique multipotent cells in adult human mesenchymal cell populations. *Proc Natl Acad Sci U S A*. 2010;107:8639-8643. doi:10.1073/pnas.0911647107
